# Prevalence of thoracic pain in patients with chronic obstructive pulmonary disease and relationship with patient characteristics: a cross-sectional observational study

**DOI:** 10.1186/s12890-016-0210-8

**Published:** 2016-04-06

**Authors:** D. J. A. Janssen, E. F. M. Wouters, Y. Lozano Parra, K. Stakenborg, F. M. E. Franssen

**Affiliations:** Department of Research & Education, CIRO, Centre of expertise for chronic organ failure, PO Box 4009, 6080 AA HAELEN, Hornerheide 1, 6085 Horn, NM The Netherlands; Centre of Expertise for Palliative Care, Maastricht University Medical Centre (MUMC+), Maastricht, The Netherlands; Department of Respiratory Medicine, Maastricht University Medical Centre (MUMC+), Maastricht, The Netherlands

**Keywords:** COPD, Pain, Lung hyperinflation, Health status, Respiratory disease

## Abstract

**Background:**

Objectives of this study were to evaluate the prevalence of thoracic pain in patients with chronic obstructive pulmonary disease (COPD) and its relationship with Forced Expiratory Volume in the first second (FEV_1_), static hyperinflation, dyspnoea, functional exercise capacity, disease-specific health status, anxiety, and depression.

**Methods:**

This cross-sectional observational study included patients with COPD entering pulmonary rehabilitation. Participants underwent spirometry, plethysmography, and measurement of single breath diffusion capacity. Pain was assessed using a multidimensional, structured pain interview. In addition, dyspnoea severity (Modified Medical Research Council Dyspnoea Scale (mMRC)), functional exercise capacity (six-minute walking distance (6MWD)), disease-specific health status (COPD Assessment Test (CAT)), and symptoms of anxiety and depression (Hospital Anxiety Depression Scale (HADS)) were recorded.

**Results:**

55 of the included 67 participants reported chronic pain (82.1 %). 53.7 % had thoracic pain. After considering multiple comparisons, only younger age and worse CAT scores were related with the presence of thoracic pain (*p* = 0.01). There were no relationships between thoracic pain and FEV_1_, static lung hyperinflation, diffusion capacity, mMRC score, 6MWD, anxiety or depression.

**Conclusion:**

Thoracic pain is highly prevalent in COPD patients and is related to impaired disease-specific health status, but there is no relationship with FEV_1_, static hyperinflation, dyspnoea severity or functional exercise capacity.

## Background

Chronic obstructive pulmonary disease (COPD) is a lung disease characterized by persistent airflow limitation and chronic inflammation of the airways that is usually progressive [1]. It is the third leading cause of death worldwide [2]. Comorbidities, such as cardiovascular disease, depression, anxiety and obesity contribute to the severity of COPD [3]. COPD itself can significantly decrease quality of life [4] and the frequently associated comorbidities may further impair quality of life [1].

Patients with COPD report multiple symptoms, which are not limited to typical COPD complaints such as dyspnoea and coughing, but also include muscle weakness, sleeplessness, low mood and pain [5]. Pain, defined as an unpleasant sensory and emotional experience associated with actual or potential tissue damage [6], is indeed reported in 21–77 % of patients with COPD [7]. According to Lee et al. [8] higher pain intensity in COPD patients is associated with more dyspnoea, depression, anxiety and poor quality of life. Roberts et al. [9] showed that patients with COPD might report thoracic pain or painful respiration. However, data about the prevalence of thoracic pain in COPD are scarce. A Norwegian study showed that 36 % of outpatients with moderate to very severe COPD entering pulmonary rehabilitation (PR) reported thoracic pain [10].

Symptom burden is an important determinant of disease-specific health status and therefore optimal symptom management is of major importance [11]. Thus, understanding the causal mechanism of thoracic pain in COPD is essential for optimal treatment. A systematic review suggested that pain and dyspnoea are related in COPD [7]. More specifically, Bentsen et al. [10] hypothesized that thoracic pain and dyspnoea could be related. This is supported by the fact that primary and accessory muscles of breathing in patients with COPD are frequently used to manage their breathlessness [10]. Hyperinflation plays an important role in the development of dyspnoea [12]. However, to date the relationship between hyperinflation and thoracic pain in patients with COPD has not been studied.

Therefore, the objectives of this study were: 1) to study the prevalence of thoracic pain in patients with COPD entering PR; and 2) to explore the relationship between thoracic pain and patient characteristics, including Forced Expiratory Volume in the first second (FEV_1_), static hyperinflation, dyspnoea, functional exercise capacity, disease-specific health status, anxiety, and depression in patients with COPD. We hypothesized a priori that thoracic pain in patients with COPD is highly prevalent, is related to static lung hyperinflation, and is associated with impairment in disease-specific health status.

## Methods

### Study design

The current study is a prospective cross-sectional observational study including patients with COPD entering PR. The medical ethical committee of the Maastricht University Medical Centre, Maastricht, the Netherlands concluded that while all tests, except a structured pain interview, were done as part of the clinical routine, the study did not fall under the Medical Research Involving Human Subjects Act and therefore did not require medical ethical approval. The local Institutional Review Board of CIRO approved the study.

### Participants

Eligible participants were patients with a diagnosis of COPD according to the Global strategy for the diagnosis, management, and prevention of chronic obstructive pulmonary disease (GOLD) [1] who were referred for PR to CIRO, a centre of expertise for chronic organ failure in Horn, the Netherlands [13]. Participants were excluded if they were unable to understand Dutch or had lung cancer as primary diagnosis. All participating patients gave written informed consent. Participants were recruited between September 2014 and November 2014.

### Measurements

As part of the regular three-day assessment performed before the PR programme, the following parameters were recorded: demographics, past medical history (Charlson comorbidity index) [14], smoking history, body mass index (BMI) and use of medication. Severity of dyspnoea was assessed by the Modified Medical Research Council Dyspnoea Scale (mMRC) [15]. FEV_1_ and Forced Vital Capacity (FVC) were measured using post-bronchodilator spirometry. Severity of airflow limitation was defined according to GOLD (1). Whole-body plethysmography was performed to measure Functional Residual Capacity (FRC), Residual Volume (RV), and Total Lung Capacity (TLC). Static hyperinflation was defined as transcendence above 120 % of the expected values from one or more of these three parameters. Single breath diffusion capacity for Carbon monoxide (TLCO) and Transfer Coefficient (KCO) were measured [16]. Patients also performed two six-minute walk tests and the test with the longest six-minute walk distance (6MWD) was used for further analysis [17]. Disease-specific health status was assessed using the COPD Assessment Test (CAT) (score ranging from 0 (best) to 40 points (worst)) [18]. Symptoms of anxiety and depression were recorded using the Hospital Anxiety and Depression Scale (HADS) [19]. The HADS consists of an anxiety subscale (HADS-A) and depression subscale (HADS-D), each ranging from 0 (best) to 21 points (worst).

In addition to the tests performed in the clinical routine, pain was assessed using a validated Dutch multidimensional, structured pain interview [20, 21]. This instrument includes items of the McGill Pain Questionnaire and the Brief Pain Inventory (BPI) [22, 23]. Prior to assessment of pain, participants were asked: ‘Are you generally bothered by pain?’ If they responded ‘yes’, pain interviews were completed. If they responded ‘no’, participants did not complete the pain interview. Participants were asked to mark the location of their pain using the body outline diagram of the BPI. Interview questions included the severity and character of pain and pain treatment. Participants were also asked to rate their pain on a numeric rating scale (NRS) ranging from 0 (no pain) to 10 (worst pain), at the time of answering the questionnaire, during the previous week, at its worst and at its least. One assessor (Y.L.P.) completed all pain interviews. Interviews took place on day one or two of the three-day assessment and lasted about 10–15 min.

### Statistics

Data were analysed using SPSS for Mac version 21.0. All continuous data were tested for normality and are shown as mean (SD). Frequencies were used to describe the prevalence of pain generally and the prevalence of pain in each location. Demographic and clinical characteristics were compared between participants with and without thoracic pain. Chi square tests were used to compare categorical data between these two groups. Independent sample T-tests or Mann–Whitney U tests were used for continuous variables, according to the variable distribution. The correlations between the severity of thoracic pain and FEV_1_, FRC, RV, and TLC as percentages of their predicted values, was determined using Pearson correlation coefficients and are shown in scatter plots. Because of multiple comparisons, a *p*-value ≤0.01 was considered statistically significant.

## Results

### Patient characteristics

In total, 72 COPD patients were asked to participate in the study and 69 provided informed consent (response rate 95.8 %). Two patients had a FEV_1_/FVC >0.7 and were therefore excluded. The final study population consisted of 67 participants.

More than half of the participants were male (59.7 %) and average age was 64.9 years. (Table 1) Seven participants (10.4 %) were classified as COPD grade I, 25 as GOLD grade II (37.3 %), 23 as GOLD grade III (34.3 %) and twelve as GOLD grade IV (17.9 %). Diabetes mellitus, ischemic heart disease and peripheral artery disease were the most frequently reported comorbidities. Lung volumes were measured in 64 patients and static lung hyperinflation was present in 47 participants (73.4 %).

**Table 1:** Patient characteristics

	Total group (*n* = 67)	Patients with thoracic pain (*n* = 36)	Patients without thoracic pain (*n* = 31)	*p*-value
Demographics				
Male	40 (59.7 %)	17 (47.2 %)	23 (74.2 %)	0.05
Age, years	64.9 (10.2)	62.0 (9.2)	68.2 (10.5)	0.01
Lung function				
FEV_1_, l	1.3 (0.6)	1.4 (0.8)	1.2 (0.4)	0.17
FEV_1_, % pred	50.0 (20.3)	52.8 (23.3)	46.7 (16.0)	0.21
Tiffeneau index (%)	39.0 (13.2)	40.2 (13.7)	37.6 (12.7)	0.44
FRC^a^, l	4.5 (1.1)	4.3 (0.9)	4.8 (1.3)	0.09
FRC^a^, % pred	143.5 (35.0)	144.0 (35.8)	142.9 (34.5)	0.90
RV^b^, l	3.5 (1.0)	3.4 (0.9)	3.6 (1.1)	0.26
RV^b^, % pred	157.5 (50.4)	161.2 (51.2)	153.3 (50.1)	0.54
TLC^b^, l	6.7 (1.3)	6.6 (1.1)	6.8 (1.5)	0.48
TLC^b^, % pred	113.9 (17.9)	118.0 (16.6)	109.2 (18.3)	0.05
TLCO^b^, mmol/min/kPa	4.5 (2.2)	4.9 (2.6)	4.0 (1.4)	0.23^e^
TLCO^b^, % pred	54.0 (21.4)	58.4 (24.7)	49.1 (16.0)	0.15^e^
KCO^b^	0.9 (0.3)	1.0 (0.3)	0.9 (0.3)	0.30
KCO^b^, % pred	67.8 (23.1)	68.0 (24.8)	67.5 (21.5)	0.93
Clinical characteristics				
BMI, kg/m^2^	27.1 (5.7)	27.9 (5.4)	26.1 (6.0)	0.19
Current Smokers	8 (11.9 %)	4 (11.1 %)	4 (12.9 %)	1.00
mMRC score, points^c^	2.6 (0.9)	2.6 (0.9)	2.5 (1.0)	0.64
CAT score, points^d^	23.3 (6.9)	25.4 (5.4)	21.0 (7.7)	0.01
HADS-A, points^d^	7.8 (4.9)	8.8 (5.0)	6.6 (4.6)	0.08
HADS-D, points^d^	8.0 (4.4)	9.1 (4.4)	6.8 (4.1)	0.03
6MWD, meters^a^	411.5 (120.6)	404.5 (121.6)	419.7 (121.1)	0.62
6MWD, % pred^a^	66.5 (19.5)	65.2 (20.1)	68.0 (19.1)	0.58
Comorbidities				
Charlson comorbidity index score, points)	1.9 (1.3)	1.7 (1.1)	2.2 (1.6)	0.11^e^
IHD	11 (16.4 %)	5 (13.9 %)	6 (19.4 %)	0.79
PAD	12 (17.9 %)	6 (16.7 %)	6 (19.4 %)	1.0
DM	10 (14.9 %)	4 (11.1 %)	6 (19.4 %)	0.55

Data are shown as mean (SD) or number (%).^a^ *n* = 63. ^b^ *n* = 64. ^C^ *n* = 66. ^d^ *n* = 65. ^e^Non-parametric tests were used because of skewed data

*Abbreviations*: *FEV*_*1*_ Forced Expiratory Volume within the first second, *FEV*_*1*_ *% pred* FEV_1_ as percentage of its predicted value, *FRC* Functional Residual Capacity, *FRC % pred* FRC as percentage of its predicted value, *RV* Residual Capacity, *RV % pred* RV as percentage of its predicted value, *TLC* Total Lung Capacity, *TLC % pred* Total Lung Capacity as percentage of its predicted value, *TLCO* Transfer factor Carbo Monoxide, *TLCO % pred* TLCO as percentage of its predicted value, *KCO* Transfer Coefficient, *KCO % pred* KCO as percentage of its predicted value, *BMI* Body Mass Index, *mMRC* modified Medical Research Council Dyspnoea Scale, *CAT* COPD Assessment Test, *HADS-A* Hospital Anxiety Depression Scale, anxiety subscale, *HADS-D* Hospital Anxiety Depression Scale, depression subscale, *6MWD* six minute walking distance, *IHD* Ischemic Heart Disease, *CHF* Congestive Heart Failure, *PAD* Peripheral Artery Disease, *DM* diabetes mellitus

### Pain prevalence

**Fig. 1:**
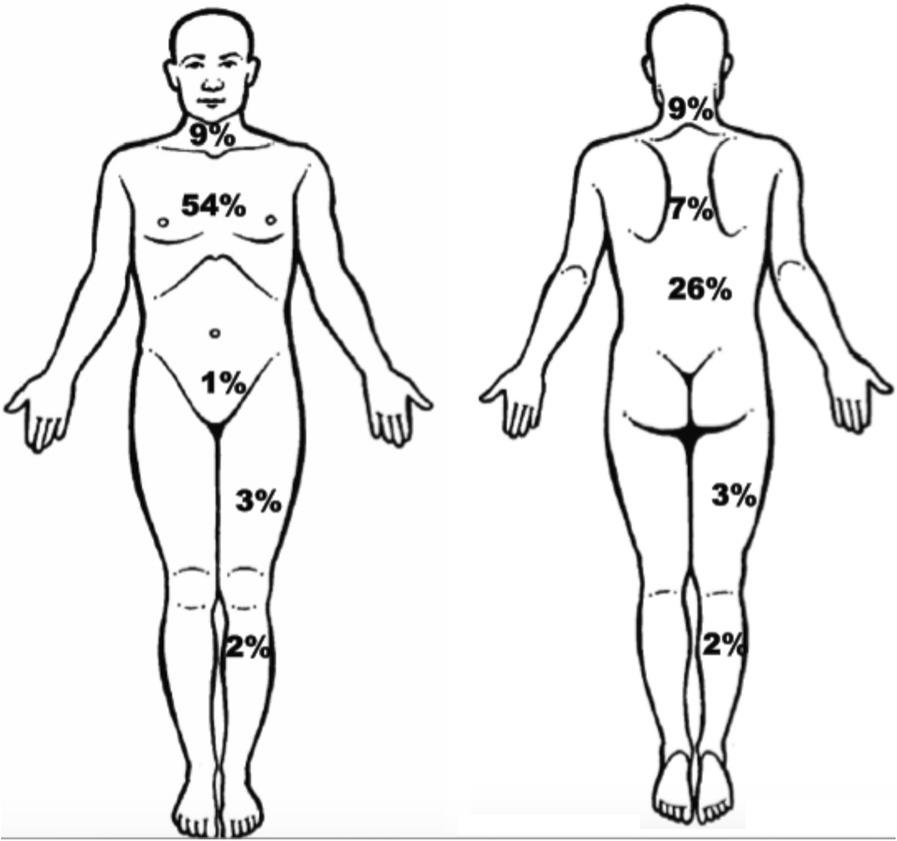
Percentages of patients with COPD reporting chronic pain in different locations. Reproduced with permission [21]

Pain was reported by 55 participants (82.1 %). Thoracic pain was reported by 36 participants (53.7 %) (Fig. 1). Of these, 27 (75.0 %) only reported thoracic pain. Further common locations for pain were: neck , shoulder blades and lower back (Fig 1). A combination of thoracic pain and pain between shoulder blades was reported by 1 participant (1.5 %), while a combination of thoracic pain and lower back pain was reported by 7 participants (10.4 %). Participants described their thoracic pain mostly as pressing pain (51.4 %).

### Characteristics of participants with or without thoracic pain

Participants with thoracic pain were younger (Table 1). FEV_1_and FEV_1_/FVC were comparable between participants with or without thoracic pain. Diffusion capacity parameters were also comparable between these two groups. mMRC scores were comparable between participants with and without thoracic pain. 6MWD was comparable for patients with and without thoracic pain. CAT score of participants with thoracic pain was significantly higher (worse) compared to participants without thoracic pain. Differences in HADS-A, HADS-D scores did not reach the level of statistical significance. Use of pain medication was comparable between patients with and without thoracic pain (Table 2).

**Table 2:** Pain medication use and pain scores, stratified for presence of thoracic pain

Pain medication use	Total group (*n* = 67)	Patients with thoracic pain (*n* = 36)	Patients without thoracic pain (*n* = 31)	*p*-value
Patients using pain medication	26 (38.8 %)	10 (27.8 %)	16 (51.6 %)	0.08
Paracetamol	15 (22.4 %)	7 (19.4 %)	8 (25.8 %)	0.74
NSAID	10 (14.9 %)	3 (8.3 %)	7 (22.6 %)	0.20
Mild opioids	1 (1.5 %)	1 (2.8 %)	0 (0.0 %)	1.0
Strong opioids	3 (4.5 %)	1 (2.8 %)	2 (6.5 %)	0.89
Pain scores	Total group (*n* = 55)	Patients with thoracic pain (*n* = 36)	Patients with other than thoracic pain (*n* = 19)	*p*-value
NRS, current pain, points	1.9 (2.8)	1.9 (2.9)	1.9 (2.6)	0.97
NRS, pain last week, points	3.4 (3.1)	2.9 (3.0)	4.4 (3.0)	0.08
NRS, min. bothering pain, points	1.1 (1.8)	1.2 (2.0)	0.9 (1.3)	0.96^a^
NRS, max. bothering pain, points	7.1 (1.8)	7.0 (1.8)	7.3 (1.8)	0.61

Data are shown as mean (SD) or number (%). ^a^Non-parametric tests were used because of skewed data

*Abbreviations*: *NSAID* Non Steroidal Anti Inflammatory Drugs, *NRS* Numeric rating scale

### Relationship between thoracic pain and static hyperinflation

**Fig. 2:**
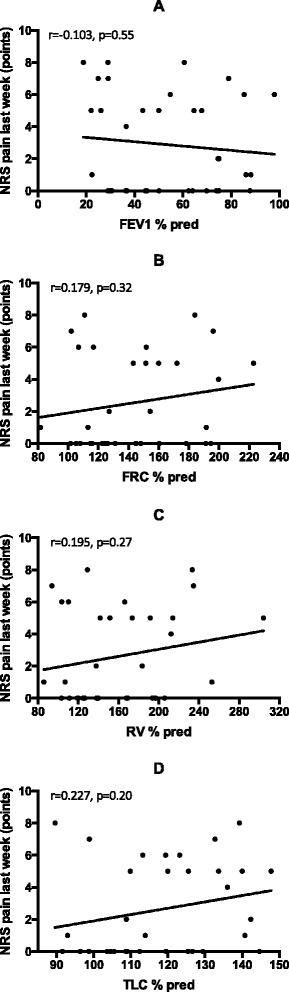
The correlation between the severity of pain in the last week and FEV_1_ % pred. (panel **a**, *n* = 36); FRC % pred. (panel **b**, *n* = 33), RV % pred. (panel **c**, *n* = 34), TLC % pred. (panel **d**, *n* = 34) among patients with thoracic pain

There were no differences in static lung volumes between the two groups. Association between severity of thoracic pain and lung function parameters is shown in Fig. 2. None of the correlations shown in either figure was found to be significant.

## Discussion

This study shows that more than half of the patients with COPD referred for PR report thoracic pain. Participants with thoracic pain are younger and report a more severely impaired disease-specific health status compared to participants without thoracic pain, despite a comparable degree of airflow limitation and other clinical characteristics.

### Pain prevalence

More than half of the participants entering PR reported thoracic pain. In addition neck, shoulder blade and lower back pain were also reported. The prevalence of thoracic pain was higher in the current study than was previously shown in outpatients with COPD attending a PR program, despite a comparable degree of airflow limitation [10] Borge et al. [24] also showed a high prevalence of pain in general, but a lower prevalence of thoracic pain than in the present study. This lower prevalence of thoracic pain may be explained by the fact that Borge et al. [24] included hospital outpatients, while the current study included patients referred for PR.

### Characteristics of participants with or without thoracic pain

This study confirmed our hypothesis that thoracic pain in COPD patients is related to impaired disease-specific health status. Moreover, differences in CAT scores between patients with and without thoracic pain were more than twice the minimum clinical importance difference [18, 25, 26]. The qualitative study from Lohe et al. [27] also showed a significant impact of pain on quality of life among patients with COPD. In fact, pain caused some patients to be homebound or even bedbound.

We could not confirm our hypothesis that static hyperinflation is related with thoracic pain in COPD. In fact, this study showed no significant differences in static hyperinflation between patients with and without thoracic pain, and no correlations between severity of thoracic pain and static hyperinflation. Thoracic pain was also not explained by differences in severity of airflow limitation or diffusion capacity. Some authors suggested that comorbidities may be responsible for pain in COPD [7]. We did not find a relationship between the Charlson comorbidity score and the presence of thoracic pain. Moreover, the literature concerning the role of comorbidities in the presence of pain in COPD remains conflicting [7]. Therefore, mechanisms underlying thoracic pain in patients with COPD remain unclear.

In the present study, prevalence of thoracic pain was higher in younger participants. Habraken et al. [28] previously found in a qualitative study that older patients are less likely to report their symptoms and tend to adapt to their situations or attribute their symptoms to getting older.

The present study showed a trend towards female participants being more likely to report thoracic pain. Higher symptom burden among female participants with COPD has been reported in earlier studies. Indeed, female patients with COPD reported more severe dyspnoea, experienced more physical and mental limitations and had lower health status scores [29, 30]. Lamprecht et al. [31]. stated that female patients, even with the same degree of lung function impairment, more often report symptoms such as dyspnoea and coughing.

We did not find statistically significant differences in scores for anxiety and depression between patients with and without thoracic pain. This may be due to the limited sample size of our study. Indeed, participants with thoracic pain reported clinically relevant [32] higher scores HADS anxiety and HADS depression scores than participants without thoracic pain. Lohne et al. [27] previously described the relationship between psychological symptoms and thoracic pain as the ‘vicious COPD circle’, stating that incomprehensible pain could lead to sleep disorders, anxiety and depression, which then results in worsening of upper body pain.

Earlier studies showed a positive relationship between pain and dyspnoea severity [33, 34] Bentsen et al. [34] hypothesized earlier that airflow limitation increases respiratory muscle work resulting in fatigue, exhaustion and pain. In the current study we did not find a difference in mMRC scores between the two groups. This may be caused by the fact that the mMRC is a situational measure, which is limited responsive to change [35]. Future studies should consider including a NRS or Visual Analogue Scale (VAS) to measure the intensity of breathlessness. A recent study including 266,000 Medicare Managed Care recipients showed that dyspnea and pain commonly occur together [36]. In fact, participants with dyspnea had a considerably higher prevalence of pain, including thoracic pain. However, Clark et al. [36] found no evidence that underlying diseases, such as COPD were completely responsible for the co-occurrence of breathlessness and pain. Moreover, the authors hypothesized that physical deconditioning might be the explanation for the relationship between breathlessness and pain. Both symptoms could be the results of the same underlying process [36].

### Treatment of pain in patients with COPD

Evidence now shows that pain, including thoracic pain, is highly prevalent among patients with COPD, and it has a major impact on disease-specific health status. As a consequence, systematic assessment and treatment of thoracic pain should be part of standard clinical care for patients with COPD to minimize symptom distress and optimize disease-specific health status. Nevertheless, only 39 % of the patients in our study were using pain medication, while 82 % of the patients reported pain. Moreover, worst reported pain scores were about 7 points, reflecting severe pain [37]. Under treatment of pain has been reported before among patients with COPD [5]. A possible explanation for pain under treatment is that patients with COPD may not actively express a wish for help for their symptoms, because they adapt to their situation or believe that there are no possibilities to improve their situation [28]. A second explanation for under treatment of pain in COPD might be the lack of knowledge concerning causal mechanisms of pain in COPD and therefore lack of causal treatment. In the light of the above described hypothesis of Clark et al. [36], it might be reasonable to assume that PR, which has been shown to improve physical functioning and decreases dyspnea severity [13], could also reduce pain in patients with COPD. If future studies could confirm this hypothesis, PR might be a non-pharmacological intervention to treat pain in patients with COPD.

### Methodological considerations

This is the first study that specifically investigates the relationship between thoracic pain and multiple clinical characteristics in a well-characterized sample of COPD patients. Nevertheless, several limitations need to be considered in interpreting the results of the study. First, the present study only included a limited number of participants, which may result in underpowerment. Second, the present study did not include a control group without COPD. Therefore, it was not possible to verify whether thoracic pain symptoms are more common in patients with COPD than in non-COPD controls. Future studies should include a non-COPD control group to explore whether and to what extent thoracic pain is related with COPD. Third, only patients referred for PR were included which limits the generalizability of the results. Finally, this study was cross-sectional, whereas longitudinal daily recording of thoracic pain may be more reliable.

## Conclusions

The current study was not able to establish an association between thoracic pain and clinical characteristics in COPD, as no correlations were found with airflow limitation, static hyperinflation or dyspnoea. However, thoracic pain proved to be very common and correlated with more severely impaired disease-specific health status. In order to optimally treat symptoms and improve disease-specific health status, awareness about the high prevalence of thoracic pain and its clinical impact must be increased among (respiratory) physicians. Assessment of thoracic pain should be integrated in COPD management programmes, including PR. Future studies should explore the mechanisms underlying thoracic pain in COPD. Longitudinal studies using pain logs, including pain intensity as well as timing of pain, might be useful. Laboratory studies could shed more light on for example the relationship between exercise and thoracic pain. Further, the possible role of deconditioning should be explored. In addition, randomized-controlled trials should study the effects of pharmacological as well as non-pharmacological interventions, including PR, on thoracic pain in COPD. Finally, these studies need to explore whether treatment of pain results in improvement of disease-specific health status.

### Ethics approval and consent to participate

The medical ethical committee of the Maastricht University Medical Centre, Maastricht, the Netherlands concluded that while all tests, except a structured pain interview, were done as part of the clinical routine, the study did not fall under the Medical Research Involving Human Subjects Act and therefore did not require medical ethical approval. The local Institutional Review Board of CIRO approved the study. All participating patients gave written informed consent.

## Availability of data and materials

Data are available upon request.

## Abbreviations

6MWD: six-minute walk distance
BMI: body mass index
BPI: brief pain inventory
CAT: COPD assessment test
COPD: chronic obstructive pulmonary disease
FEV_1_: forced expiratory volume in the first second
FRC: functional residual capacity
FVC: forced vital capacity
GOLD: global strategy for the diagnosis, management, and prevention of chronic obstructive pulmonary disease
HADS: hospital anxiety and depression scale
HADS-A: hospital anxiety and depression scale, anxiety subscale
HADS-D: hospital anxiety and depression scale, depression subscale
KCO: transfer coefficient
mMRC: Modified Medical Research Council Dyspnoea Scale
NRS: numeric rating scale
PR: pulmonary rehabilitation
RV: residual volume
TLC: total lung capacity
TLCO: single breath diffusion capacity for carbon monoxide
